# La tumeur de Buschke-Löwenstein

**DOI:** 10.11604/pamj.2016.25.176.10622

**Published:** 2016-11-18

**Authors:** Hakima Elmahi, Fatima Zahra Mernissi

**Affiliations:** 1 Service de Dermatologie, CHU Hassan II, Fès, Maroc

**Keywords:** Tumeur de Buschke Lowenstein, condylome acuminé géant, papillomavirus humain, Buschke Löwenstein tumor, giant condylomata acuminata, human papillomavirus

## Image en médecine

La tumeur de Buschke-Lownestein (TBL) ou condylome acuminé géant, est une maladie sexuellement transmissible à papillomavirus humain (HPV) le plus souvent de type 6 et/ou 11, rare dont la fréquence est estimée à 0,1% de la population générale avec une prédominance masculine. Elle se caractérise par son potentiel dégénératif et son caractère envahissant et récidivant après traitement. La frontière entre TBL et carcinome verruqueux reste imprécise. L'aspect histologique est en fait bénin, quoique son aspect clinique évoque plutôt le contraire. Le traitement est mal codifié et reste essentiellement chirurgical. L'éducation sexuelle et le traitement précoce des lésions condylomateuses permettent d'améliorer le pronostic. Nous rapportons le cas d'un patient âgé de 51 ans, ayant comme antécédent une notion de vagabondage sexuel, qui consulte pour une tumeur périnéo-scrotale évoluant depuis 30 ans. L'examen clinique notait la présence d'une tumeur dyschromique, infiltrée, papillomateuse en chou-fleur, ano-périnéale et scrotale, fétide et indolore. Les aires ganglionnaires étaient libres. Les sérologies VIH, syphilitique et des hépatites B et C étaient négatives. L'examen histologique d'un prélèvement biopsique a mis en évidence une hyperplasie épithéliomateuse faite d'un revêtement malpighien acanthosique, papillomateux, surmonté par une hyperkératose parakératosique avec présence de koïlocytes signant l'infection par le HPV, il n'a pas été noté d'atypie cellulaire. Le diagnostic de tumeur de Buschke-Löwenstein a été retenu et le patient a été adressé au service de chirurgie urologique où une exérèse large a été réalisée. L'évolution a été bonne sans récidive avec un recul de 2 ans.

**Figure 1:**
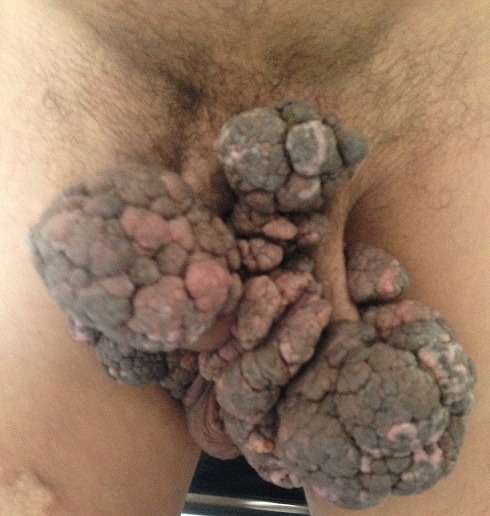
Tumeur périnéo-scrotale papillomateuse en chou-fleur

